# A Smartphone Indoor Localization Algorithm Based on WLAN Location Fingerprinting with Feature Extraction and Clustering

**DOI:** 10.3390/s17061339

**Published:** 2017-06-09

**Authors:** Junhai Luo, Liang Fu

**Affiliations:** School of Electronic Engineering, University of Electronic Science and Technology of China, Chengdu 610073, China; fulianguestc@std.uestc.edu.cn

**Keywords:** indoor localization, received signal strength, AP selection, kernel principal component analysis, affinity propagation clustering

## Abstract

With the development of communication technology, the demand for location-based services is growing rapidly. This paper presents an algorithm for indoor localization based on Received Signal Strength (RSS), which is collected from Access Points (APs). The proposed localization algorithm contains the offline information acquisition phase and online positioning phase. Firstly, the AP selection algorithm is reviewed and improved based on the stability of signals to remove useless AP; secondly, Kernel Principal Component Analysis (KPCA) is analyzed and used to remove the data redundancy and maintain useful characteristics for nonlinear feature extraction; thirdly, the Affinity Propagation Clustering (APC) algorithm utilizes RSS values to classify data samples and narrow the positioning range. In the online positioning phase, the classified data will be matched with the testing data to determine the position area, and the Maximum Likelihood (ML) estimate will be employed for precise positioning. Eventually, the proposed algorithm is implemented in a real-world environment for performance evaluation. Experimental results demonstrate that the proposed algorithm improves the accuracy and computational complexity.

## 1. Introduction

In recent years, with the rapid development and popularization of mobile Internet, the demand for Location-Based Services (LBSs) [1] has gradually increased, which makes it feasible to obtain and utilize location information through smartphones, tablets and other mobile terminals. LBSs have become indispensable in our lives. Meanwhile, a variety of wireless network communication technologies have become universal, such as Wireless Local Area Network (WLAN) and the Fifth Generation Mobile Communication System (5G), which definitely will be prevalent in the future. The use of the wireless network in LBS also plays an important role in the application area of wireless network technology [2].

Currently, Global Position System (GPS) positioning is mainly used in an outdoor environment [3,4]. However, GPS in the indoor environment has its inevitable limitations, which can be generally summarized in the following two points. (1) As a result of the reinforced concrete blocks, the satellite signal is too weak to be considered here; (2) GPS has high power consumption on the mobile terminal. According to those above reasons, GPS cannot work very well in indoor environments.

Several papers have studied typical indoor location methods, like Infrared (IR) [5,6], Ultrasound (US) [7,8] and Radio Frequency Identification (RFID) [9,10]. These methods have a high requirement for the environment and need additional hardware devices. With the WLAN developing, hundreds of APs are deployed in the buildings. Meanwhile, RSS-based localization algorithms have been extensively studied as an inexpensive solution for positioning [11,12,13]. Compared with other WLAN-based algorithms, like Time-Of-Arrival (TOA) [14] or Angle-Of-Arrival (AOA) [15], RSS fingerprint positioning technology need not estimate too many parameters, which could be against indoor multipath propagation effectively and improve the accuracy of indoor positioning. Furthermore, without any additional hardware, RSS can be easily obtained from APs whose positions do not have to be known in advance. Therefore, this technology is more universal.

The major challenge in indoor localization is accuracy because of uncertain factors, such as the fluctuation of the RSS signal. Some positioning methods have been raised to solve this problem, like KNN [16] and Weighted K-Nearest-Neighbor (WKNN) [17]. They are used to calculate the RSS values of the K groups that are closest to the real-time RSS samples in the fingerprint database. The position coordinates of K groups are used to estimate user’s location. This kind of method is easily implemented, but hardly gets accurate results. It is more suitable for simple indoor positioning. Another solution is to adopt the mathematical statistics methods, like Bayesian theory [18] and maximum likelihood [19]. They establish the fingerprint mapping to conclude the probability of the user’s location. Methods based on statistics are more accurate than KNN and WKNN, but in a real circumstance, the RSS distribution may differ from the theoretical analysis, and it requires collecting many training samples to get a more accurate signal distribution model.

The stable AP selection algorithm is studied to select APs that are useful for positioning. Due to issues like obstruction and the multipath effect, the RSS signals detected at RPs are extremely unstable. Thus, saving all RSS signals in the fingerprint database influences the accuracy of the positioning system [20]. This paper exploits the theory of KPCA to extract nonlinear features and reduce complexity. It is found that the impacts on the fingerprint positioning accuracy often have a great relationship with the data source in the fingerprint collection phase. Different RSS values of various wireless routers contribute to the estimation of the position. Even some RSS values exert opposite effects on the position judgment. If the useful data components of the data source are determined, the positioning system accuracy will be improved. Meanwhile, KPCA can also reduce the dimension of the fingerprint vector to improve the processing efficiency of the fingerprint localization algorithm. To get the optimal clustering results, we also use the theory of the APC algorithm to improve the clustering effect and reduce the probability of the improper initial clustering center in artificial selection [21,22]. This algorithm is based on the similarity between two different data points, which does not require a special clustering number in advance. On the contrary, it regards all data points as possible exemplars. It is based on distance measurement and automatically classifies similar points into the same cluster.

This paper proposes a smartphone indoor localization algorithm that consists of two stages: the offline stage and the online stage. The offline stage mainly accomplishes fingerprint information collection and acquires RSS, which can adequately describe the spatial characteristics of the regional positioning database. The online stage compares the fingerprint of Reference Points (RPs) in the database with the RSS, which is measured at the particular location in a timely manner to estimate the user’s location. In the offline phase, the RSS value, which is of the APs at different RPs, is optimized through the stable AP selection algorithm and the KPCA method. Afterward, the RPs are classified by the APC algorithm. In the online phase, the mobile device collects RSS values, which are selected to match with the cluster, and then uses ML to estimate the user’s location. Taking into account the privacy of the users’ locations, the positioning process is usually finished on the terminal device. In view of computing power, storage capacity and limited energy of the mobile terminal, the purpose of this paper is to design an indoor positioning algorithm based on location fingerprinting to improve the positioning accuracy and verify the effectiveness of this algorithm.

The remainder of this paper is organized as follows. The particular positioning algorithm is presented and introduced in Section 2. This algorithm is analyzed and evaluated in Section 3. Section 4 concludes the paper.

## 2. Modeling of Positioning System

Due to the complexity of the indoor environment, many difficulties arise in predicting wave propagation in this case. For the indoor positioning environment, the complex room structure and the crowd will have a high impact on the wireless signal transmission, resulting in the multi-path propagation effect.

This paper proposes a positioning system that is designed to optimize the accuracy, computational complexity and energy consumption. Because of hundreds of RPs and APs, the large consumption of the system mainly occurs in the calculation during the position estimation process. Therefore, this paper aims to decrease the database size and the data dimensions while performing position matching. It is obtained by reducing the number of RPs and APs, which are reserved in the database for excellent positioning. In the position-estimation phase, the mobile terminal adopts cluster matching and the ML estimate algorithm for precise localization, so as to achieve the reduction of energy consumption and high accuracy in positioning terminals.

**Figure 1:**
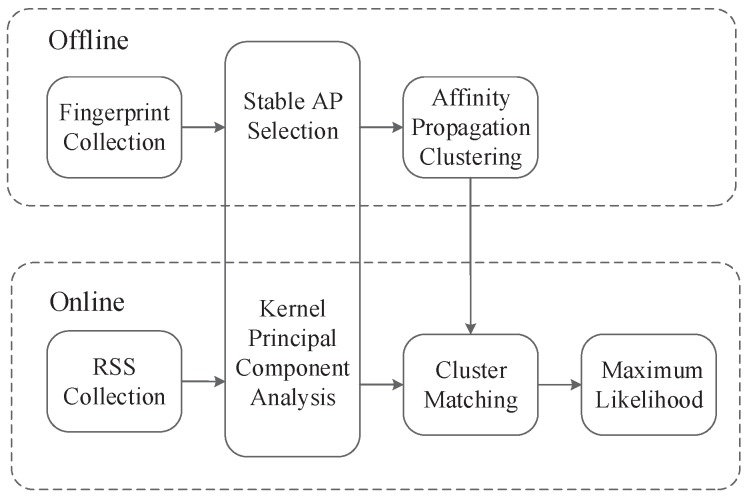
Indoor localization model.

The model of the WLAN indoor positioning system based on location fingerprinting is shown in Figure 1, which contains two stages: offline training and online positioning. In the offline phase, some RPs are selected in the location area, and RSS time series are measured from each AP at each RP, which is utilized to create the location fingerprint database. After the completion of the fingerprint collection, some APs that are not useful for positioning should be discarded in the offline AP chosen phase. In the data preprocessing phase, the correlation of the RSS information is removed by the KPCA algorithm, improving the reliability and rationality of the fingerprint. According to the similarity of the RSS vectors, all RPs are clustered into different classes, which have their exemplar by the APC algorithm. In the online phase, the RSS vector that is measured in real-time by the smartphone is matched with the fingerprinting database, and the location estimate is completed in the smartphone terminal. The AP selection and KPCA are applied to select a subset of all of the APs and to extract nonlinear features. To limit the location to one or several classes and weaken the interference of environmental factors, cluster matching matches the online RSS vector with each cluster in the fingerprint database. The ML estimate is utilized to estimate the user’s location in the indoor localization stage. Each step will be analyzed in detail in the following sections.

### 2.1. Offline Stage

#### 2.1.1. Fingerprint Collection

Fingerprint collection should be processed first in the offline phase. In this paper, the RSS and configuration information of the AP can be detected in the location area without knowing the AP information in advance, because the basic network information, including MAC address and RSS, will be broadcast by the way of radio beacons. The mobile terminal with a Wi-Fi adapter wirelessly obtains the MAC address and RSS of the AP at various RP locations. Let $\left\{\varphi_{i,j}\left(\tau \right),\tau =1,\cdots ,q,q>1\right\}$ represent the measured RSS time samples from $AP_{i}$ at an $RP_{j}$ and *q* represent the number of RSS time samples. The mean of RSS tends to be stable when q≥30 due to many factors, such as the complexity of the indoor environment and the non-line of sight propagation of the signal [23]. In this paper, the effect of antenna orientation on the positioning results is not considered so that the terminal device can always be in the same direction. The average of each AP’s *q* samples is computed by traversing all of the RPs in the location area to create a fingerprint map, which can adequately describe the characteristics of the indoor environment. It is represented by Ψ as follows:

(1) $$\Psi =\left(\begin{array}{cccc} \varphi_{1,1} & \varphi_{1,2} & \cdots & \varphi_{1,N}\\ \varphi_{2,1} & \varphi_{2,2} & \cdots & \varphi_{2,N}\\ \vdots & \vdots & \ddots & \vdots \\ \varphi_{M,1} & \varphi_{M,2} & \cdots & \varphi_{M,N} \end{array}\right)$$

where $\varphi_{i,j}=\frac{1}{q}\sum_{\tau =1}^{q}\varphi_{i,j}\left(\tau \right)$ is the mean of $AP_{i}$’s RSS at the $RP_{j}$. *M* is the number of APs that can be detected, and *N* is the number of RPs. The columns of Ψ are the mean of RSS from *M* APs at an RP, which can be referred to as follows:

(2) $$\Psi_{j}={\left(\varphi_{1,j},\varphi_{2,j},\cdots ,\varphi_{M,j}\right)}^{T}$$

To calculate the stability of each AP, it is also necessary to store the standard deviation $SD_{j}$ of each AP and the number of occurrences $FRE_{j}$. Meanwhile, the location information of RPs should be recorded in the database. Therefore, the complete location fingerprint can be expressed as $\left\{\left(x_{j},y_{j}\right);\Psi_{j};SD_{j};FRE_{j},j=1,2,\cdots ,N\right\}$, where $\left(x_{j},y_{j}\right)$ represents the coordinates of RP. In the signal-collecting process, not all of the APs can be detected. To ensure the integrity of the fingerprint, we can assign undetected APs a small default value, which is called invalid data.

In the practical positioning environment, the RSS fluctuation is quite severe due to the uncertain factors, such as multipath, diffraction, obstacles and so on. In order for more convenient clustering and instantaneous-fluctuation reduction of the measured values, the mean-smoothing filter is used to smooth the fingerprint database. The mean-smoothing filter is to assign each RP’s RSS to the average RSS of several close-to-each-other RPs, as follows:

(3) $$\varphi_{i,j}=\frac{1}{\left|m\right|}\sum_{m}\varphi_{i,m},\sqrt{{\left(x_{m}-x_{j}\right)}^{2}+{\left(y_{m}-y_{j}\right)}^{2}}\leq d$$

where $\left(x_{j},y_{j}\right)$ and $\left(x_{m},y_{m}\right)$ denote the position coordinates of the RP. *m* denotes the number of RPs satisfying the experimental requirement. *d* is a constant representing the distance, which is used to control the smoothness of the smoothing filter. It is usually slightly larger than the average distance between RPs. When the RSS is small, some of the AP measurements will be intermittent. The mean-smoothing filter can replenish some of the missing measurements.

#### 2.1.2. Stable AP Selection Alogorithm

Due to the unstable RSS signal, the RSS value, often affected by changes in the environment, is constantly changing. When the positioning area is large, not all of the APs can be measured at each RP due to the coverage-range limitation of the radio-frequency signal. At an RP, if an unmeasured AP is detected in the online phase, it will result in a vast distance between the RSS vectors online and offline because it is represented by a small default value in the database. If the AP is used for positioning, a large positioning error will appear. Therefore, when the RP number with invalid RSS values accounts for a large proportion, AP should be removed. To reduce the computational complexity and enhance the positioning stability, the stable AP selection algorithm is proposed to deal with this problem. In the positioning area, the APs’ signals received at each sampling point are not exactly equal, which are expressed as $\left\{AP_{1},AP_{2},\cdots ,AP_{M}\right\}$. At the location *L*, the qsamples of AP are received as $\left\{RSS_{1},RSS_{2},\cdots ,RSS_{q}\right\}$. RSS data fluctuations can be calculated in terms of variance as follows:

(4) $$SD_{AP_{i}}=\sqrt{\frac{1}{q-1}\sum_{j=1}^{q}{\left(RSS_{j}-\bar{RSS}\right)}^{2}}$$

where $\bar{RSS}$ represents the average of *q* samples, and $SD_{AP_{i}}$ reflects the magnitude of the data fluctuation. In addition, the weights are assigned to each AP depending on the frequency of AP as follows:

(5) $$FRE_{AP_{i}}=\frac{N_{i}}{\sum_{j=1}^{M}N_{j}}$$

where $N_{i}$ is the number of $AP_{i}$, and $\sum_{j=1}^{M}N_{j}$ is the total number of all of the APs. The stability of $AP_{i}$ is as follows:

(6) $$STA_{AP_{i}}=FRE_{AP_{i}}\cdot \frac{1}{SD_{AP_{i}}+\varepsilon }$$

where ε is a small positive number, preventing the denominator from being zero. The higher $STA_{AP_{i}}$ is, the more stable $AP_{i}$ is. The AP selection algorithm sorts the $STA_{AP_{i}}$ value from large to small and selects the top *K* as the location fingerprint.

#### 2.1.3. KPCA Algorithm

Feature extraction can be employed to eliminate redundancy and noise, reduce computation complexity and enhance the positioning accuracy since the storage and computation of mobile devices are limited. Traditional ways of feature extraction only analyze the linear relation among different data, such as Principal Components Analysis (PCA) [24]. Therefore, this paper applies KPCA for mapping the low-dimensional data to high-dimensional for nonlinear feature extraction, which can further increase the positioning performance compared to the traditional algorithm.

KPCA is an extension of PCA in the high-dimensional space using a kernel function. The main idea of this method is to compute the principal components of the influence variables and their weights by the eigenvectors and eigenvalues of the covariance matrix in the high-dimensional space. RSS values have a high degree of correlation and overlap with information, which will affect the positioning accuracy. Therefore, we need to use a few comprehensive indicators which are not related to each other to provide the most information.

It is assumed that χ is the original space; the kernel function $K\left(\cdot \right)$ defines a nonlinear function ϕ to map the low-dimensional data, which are linear and inseparable, into the high-dimensional feature space by nonlinear transformation, which makes the data linearly separable and simplifies mathematical calculation [25]; that is, $\phi :r\in \chi \to \phi \left(r\right)\in F$. In the feature space, the inner product of the data can be calculated by the kernel function. The relationship is represented as follows:

(7) $$K\left(r,r^{\prime }\right)=\langle \phi \left(r\right),\phi \left(r^{\prime }\right)\rangle$$

Therefore, it is not required to know the specific mapping function in the calculation. The operation in the high-dimensional space can be transformed into a kernel function in the low-dimensional space. The main principle of KPCA in the indoor positioning system is described as follows.

It is assumed that the position fingerprint data in the original space is an M-dimensional matrix Ψ. There are *N*-group fingerprints, and the nonlinear mapping function is $\phi \left(\cdot \right)$, so the transformed data $\phi \left(\Psi \right)$ can be obtained in the high-dimensional space. Assuming that $\phi \left(\Psi \right)$ is linearly separable in the current high-dimensional space, $\phi \left(\Psi \right)$ can be processed by PCA to extract nonlinear features. $\phi \left(\Psi^{*}\right)$, which satisfies $\sum_{i=1}^{N}{\varphi_{i}}^{*}=0$, can be obtained by centering matrix $\phi \left(\Psi \right)$. The covariance matrix in the high-dimensional space is represented as follows:

(8) $$C=\frac{1}{N}\sum_{i=1}^{N}\phi \left({\varphi_{i}}^{*}\right)\phi {\left({\varphi_{i}}^{*}\right)}^{T}$$

It is assumed that the D-dimensional vector $\lambda_{i}$ represents the eigenvalue of the covariance matrix C, and $w_{i}$ represents the corresponding eigenvector, where i=1,2,⋯,D, D≥M. Therefore, it can be expressed as follows:

(9) $$\phi \left(\Psi^{*}\right)\phi {\left(\Psi^{*}\right)}^{T}w_{i}=\lambda_{i}\cdot w_{i}$$

According to the linear space theorem, the eigenvector $w_{i}$ can be expressed by the linear combination of the samples $\phi \left(\varphi_{i}\right)$, which is expressed as:

(10) $$w_{i}=\sum_{i=1}^{N}\varphi_{i}\phi \left({\varphi_{i}}^{*}\right)=\phi \left(\Psi^{*}\right)\alpha$$

Equation (11) can be obtained by combining Equations (9) and (10), which is expressed as:

(11) $$\phi \left(\Psi^{*}\right)\phi {\left(\Psi^{*}\right)}^{T}\phi \left(\Psi^{*}\right)\alpha =\lambda_{i}\phi \left(\Psi^{*}\right)\alpha$$

Multiplying both sides of Equation (11) by matrix $\phi {\left(\Psi^{*}\right)}^{T}$, Equation (12) can be obtained as follows:

(12) $$\phi {\left(\Psi^{*}\right)}^{T}\phi \left(\Psi^{*}\right)\phi {\left(\Psi^{*}\right)}^{T}\phi \left(\Psi^{*}\right)\alpha =\lambda_{i}\phi {\left(\Psi^{*}\right)}^{T}\phi \left(\Psi^{*}\right)\alpha$$

According to Equation (7), the inner product in the high-dimensional space can be given by the kernel function. Therefore, Equation (12) can be expressed as follows:

(13) $$K^{2}\alpha =\lambda_{i}K\alpha$$

Equation (13) can be simplified to Equation (14) as follows:

(14) $$K\alpha =\lambda_{i}\alpha$$

where $K_{ij}=\phi {\left({\varphi_{i}}^{*}\right)}^{T}\phi \left({\varphi_{j}}^{*}\right)$. It is assumed that $I\in R^{N\times N}$, $I_{ij}=1$, i=1,2,⋯,N, j=1,2,⋯,N. Therefore, combining with the data centering, the modified kernel function matrix *K* can be expressed as follows:

(15) $$\begin{array}{c} K_{ij}=\phi {\left({\varphi_{i}}^{*}\right)}^{T}\phi \left(\varphi_{j}\right)={\left(\phi \left(\varphi_{i}\right)-\frac{1}{N}\sum_{m=1}^{N}\phi \left(\varphi_{m}\right)\right)}^{T}\left(\phi \left(\varphi_{j}\right)-\frac{1}{N}\sum_{n=1}^{N}\phi \left(\varphi_{n}\right)\right)\\ =\phi {\left(\varphi_{i}\right)}^{T}\phi \left(\varphi_{j}\right)-\frac{1}{N}\sum_{m=1}^{N}\phi {\left(\varphi_{m}\right)}^{T}\phi \left(\varphi_{j}\right)-\frac{1}{N}\sum_{n=1}^{N}\phi {\left(\varphi_{i}\right)}^{T}\phi \left(\varphi_{n}\right)+\frac{1}{N^{2}}\sum_{m,n=1}^{N}\phi {\left(\varphi_{m}\right)}^{T}\phi \left(\varphi_{n}\right)\\ =K_{ij}-\frac{1}{N}\sum_{m=1}^{N}I_{im}K_{mj}-\frac{1}{N}\sum_{n=1}^{M}K_{in}I_{nj}+\frac{1}{N^{2}}\sum_{m,n=1}^{N}I_{im}K_{mn}I_{nj} \end{array}$$

The eigenvalues and eigenvectors of the original covariance matrix can be obtained by Equation (8). The weighted matrix is composed of the eigenvectors corresponding to the eigenvalues that are arranged from large to small. It is assumed that the data sample is $\Psi_{new}$. Therefore, the transformed dataset is expressed as follows:

(16) $${\hat{\Psi }}_{new}={w_{i}}^{T}\Psi_{new}={\left(\sum_{i=1}^{N}\phi \left({\varphi_{i}}^{*}\right)\alpha_{i}\right)}^{T}\phi \left({\Psi_{new}}^{*}\right)=\alpha^{T}\phi {\left(\Psi^{*}\right)}^{T}\phi \left({\Psi_{new}}^{*}\right)$$

To solve the eigenvalues and eigenvectors of the kernel function matrix *K*, it is necessary to select an appropriate kernel function [26]. The Gaussian kernel function has favorable smoothing performance and a good ability at RSS nonlinearity. It is expressed as follows:

(17) $$K\left(x,x_{i}\right)=\exp\left(-\frac{{∥x-x_{i}∥}^{2}}{2\sigma^{2}}\right)$$

In the indoor positioning system, the specific steps of KPCA are described as follows:

1. Ψ is the input data in the low-dimensional space, and the Gaussian kernel matrix is calculated by Equation (17).
2. The modified kernel matrix data is calculated by Equation (15).
3. Calculate the eigenvalues and eigenvectors after modifying the kernel matrix. Arrange the eigenvalues from large to small. The former K eigenvalues and the corresponding eigenvectors are selected.
4. The schemed orthogonal method is used to get the linearly independent vector group.
5. The matrix transformed by Equation (16) is stored in the fingerprint database.

#### 2.1.4. APC Algorithm

To reduce the impact of the RSS time-varying property, the APC algorithm is employed to cluster the RPs according to the similarity of RSS vectors in the fingerprint database. Therefore, those RPs with high similarity in RSS vectors are clustered into one class, whose physical positions are also close. The general clustering algorithm is to select an exemplar via numerous iterations so that the distance between the cluster centers and other members of the class can be the smallest. The APC algorithm connects all of the points in the large area and makes each node a potential exemplar. Points launch responsibility and receive availability constantly, which continue to extend the gap between the exemplar and subsidiary points until the exemplar is determined ultimately.

Assuming that $\varphi_{i}$ and $\varphi_{j}$ are the average RSS vectors of any two RPs, $s\left(i,j\right)$ indicates the similarity between $RP_{i}$ and $RP_{j}$, and the similarity function between different RPs is defined as:

(18) $$s\left(i,j\right)=-{∥\varphi_{i}-\varphi_{j}∥}^{2},\forall i\neq j\in \left\{1,2,\cdots ,N\right\}$$

The closer the spatial-distance of the sample points is, greater the numbers of the same AP signal can be searched. To a certain extent, the number of APs reflects the spatial-distance relationship among sample points. Therefore, in the calculation of the signal distance, the same APs’ quantity is introduced. The signal similarity is improved as follows:

(19) $$s\left(i,j\right)=\frac{-{∥\varphi_{i}-\varphi_{j}∥}^{2}}{m},\forall i\neq j\in \left\{1,2,\cdots ,N\right\}$$

where *m* denotes the number of identical APs between the points.

The more similar the RSS vectors between two RPs are, the greater the similarity value is. These values form an N×N similarity matrix *S*, where *N* is the total number of RPs that need to be clustered. The value $s\left(j,j\right)$ on the diagonal of the matrix *S* is called preference, denoted by $p\left(j\right)$, and used to judge whether $RP_{j}$ can become the exemplar. If the value is higher, the likelihood for the points becoming center points is greater along with a larger number of categories in the clustering results. $p\left(j\right)$ is denoted as follows:

(20) $$p=\gamma *median\left\{s\left(i,j\right),i\neq j,\forall i,j\in \left\{1,2,\cdots ,N\right\}\right\}$$

where γ denotes a constant, and it affects the number of clusters.

The APC algorithm is a continuous iteration process. The RPs transmit two kinds of information about each other, namely $r\left(i,j\right)$ (responsibility) and $a\left(i,j\right)$ (availability). They are both set to zero initially.

$r\left(i,j\right)$ denotes the confidence level of $RP_{j}$ as the exemplar of $RP_{i}$, and it is updated by Equation (21):

(21) $$r\left(i,j\right)=s\left(i,j\right)-\max_{j\neq j^{\prime }}\left\{a\left(i,j^{\prime }\right)+s\left(i,j^{\prime }\right)\right\}$$

$a\left(i,j\right)$ denotes that $RP_{i}$ selects $RP_{j}$ as the confidence center of its exemplar, and it is updated by Equation (22):

(22) $$a\left(i,j\right)=\min\left\{0,r\left(j,j\right)+\sum_{i^{\prime }\notin \left\{i,j\right\}}\max\left\{0,r\left(i^{\prime },k\right)\right\}\right\}$$

$a\left(j,j\right)$ is self-availability, which reflects the cumulative evidence for $RP_{j}$ as the exemplar, and it is calculated by Equation (23):

(23) $$a\left(j,j\right)=\sum_{i^{\prime }\neq j}\max\left\{0,r\left(i^{\prime },j\right)\right\}$$

In some cases, the algorithm cannot converge. To prevent this problem, λ is exploited to update responsibility and availability.

(24) $$r_{i}=\left(1-\lambda \right)r_{i}+\lambda r_{i-1}$$

(25) $$a_{i}=\left(1-\lambda \right)a_{i}+\lambda a_{i-1}$$

The exemplar is updated according to the value of $r\left(i,j\right)+a\left(i,j\right)$. For $RP_{i}$, if $r\left(i,j\right)+a\left(i,j\right)$ is the largest, it indicates that $RP_{j}$ is the exemplar of $RP_{i}$. Otherwise, $RP_{i}$ will be selected. The process is completed in the offline phase. *H* is denoted as the class of the exemplar set and $C_{i}$ as the class of all $RP_{j}$ class members, where j∈H.

### 2.2. Online Stage

In the offline phase, we have established the correspondence between the position coordinates of the RPs and fingerprints formed by the RSS sequence. The stable AP selection algorithm is used to select useful APs for positioning, and the KPCA is applied to reduce the original data. RPs are classified by the APC algorithm, which is regarded as the basis of the online phase.

#### 2.2.1. Cluster Matching

The purpose of cluster matching is to decrease the positioning range. It can reduce computational complexity and enhance positioning accuracy. In the online phase, the RSS vector measured by the terminal device is as follows:

(26) $$\Psi_{r}=\left[\varphi_{1,r},\varphi_{2,r},\cdots ,\varphi_{M,r}\right]$$

The terminal device calculates the similarity between each measured value $\Psi_{r}$ and each cluster and then determines the corresponding cluster to which each measured value belongs.

Two kinds of class-matching methods are provided here. One is to compare the fingerprint with the exemplar’s RSS value. The other is to search all of the members of each class to find the mean of their fingerprints and then compare them with the fingerprint. When the user’s position is at the edge of the cluster, if there is only one cluster with the largest similarity selected, the class-matching will fail. Therefore, several similar clusters with high similarity should be preserved when the class is matching.

#### 2.2.2. ML Estimate

ML is exploited to locate according to the distribution characteristics of RSS. It is assumed that there are N RPs $\left\{L_{1},L_{2},\cdots ,L_{N}\right\}$ in the area and that there are M AP RSS $\left\{s_{1},s_{2},\cdots ,s_{M}\right\}$ at RP. The location of the terminal device can be considered as the position of the reference point with the greatest posterior probability as follows:

(27) $$\left(\hat{x},\hat{y}\right)=\max_{L_{i}}P\left(L_{i}|S\right)$$

The posterior probability cannot be obtained through the distribution of RSS, which can be calculated by the Bayesian formula as follows:

(28) $$P\left(L_{i}|S\right)=\frac{P\left(S|L_{i}\right)\cdot P\left(L_{i}\right)}{P\left(S\right)}$$

where the probability that the sampling point appears at any position is equal. Therefore, $P\left(L_{i}\right)$ is uniformly distributed. $P\left(S\right)$ is a constant, which depends on the Gaussian distribution. It is estimated by mean μ and variance $\sigma^{2}$ as follows:

(29) $$\mu =\bar{S}$$

(30) $$\sigma^{2}=\frac{1}{N}\sum_{i=1}^{N}\left(S_{i}-\bar{S}\right)$$

It is assumed that the different APs’ signals measured at the position of each reference point are independent and irrelevant. The coordinates are determined by the maximum probability product as follows:

(31) $$\left(\hat{x},\hat{y}\right)=\prod_{k=1}^{M}P\left(s_{k}|L_{i}\right)$$

To estimate the position more accurately, it is possible to select a plurality of reference points with the largest probability and to assign different weights as follows:

(32) $$\left(\hat{x},\hat{y}\right)=\sum_{i=1}^{K}\hat{w_{i}}\cdot P\left(L_{i}|S\right)$$

where $\hat{w_{i}}$ is the normalized weight, and $L_{i}$ is the set of K reference points with the largest similarity.

## 3. Experimentation and Evaluation

This paper studies the RSS signal processing and positioning algorithm, which are applied to the positioning model, to verify its positioning accuracy and effectiveness in the actual Wi-Fi environment.

### 3.1. Experiment Setup

**Figure 2:**
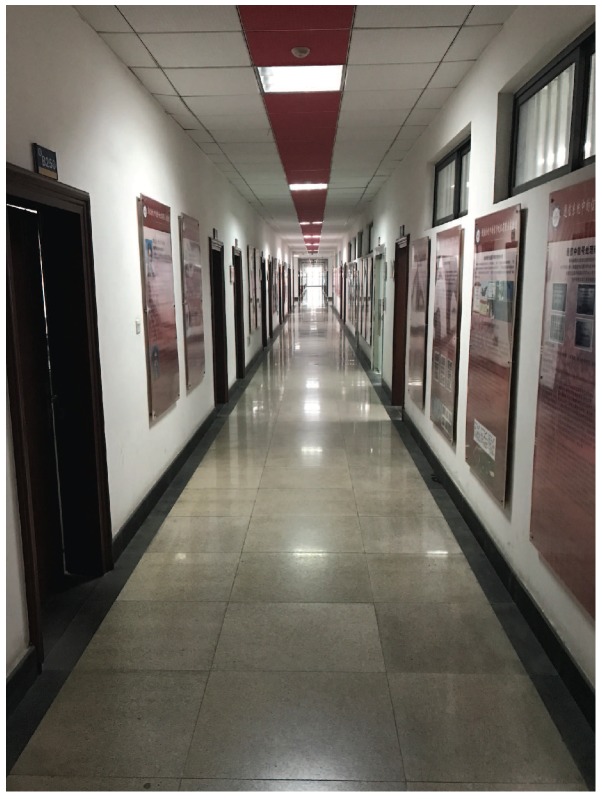
Real-world environment. There are a few rooms on both sides of the corridor, and each room has at least one AP. The user holds the mobile device for fingerprint collection and testing along the corridor.

In this paper, the experiment is conducted on the second floor of Section B of the University of Electronic Science and Technology of China. Figure 2 shows part of the experimental environment.

**Figure 3:**
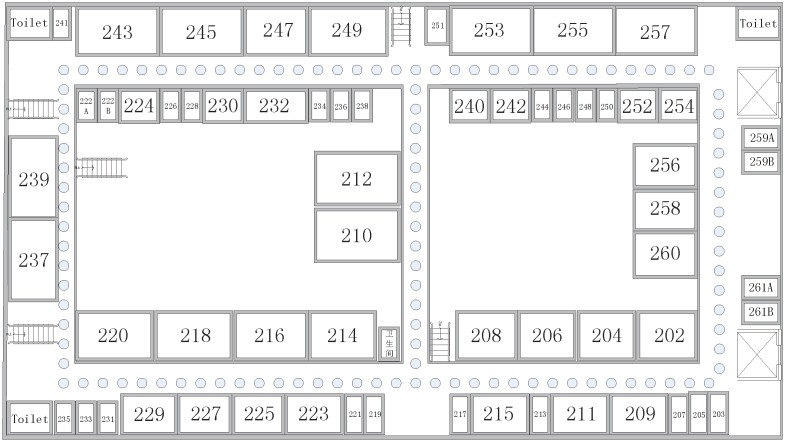
Experimental environment.

The experimental area is modeled by Microsoft Visio for an 80 m × 40 m rectangular area, as shown in Figure 3. The number of available APs and the experiment location are randomly placed without being known in advance. The positioning software is developed in Java using Android Studio and installed on the phone called MEIZU MX4 which is made in China. The results are simulated by MATLAB. A total of 115 RPs are set up within the experimental range, and data are collected at one-second intervals at each RP. The distance between adjacent RPs is approximately 2 m. At the same time, 100 test points are randomly arranged for position estimation.

### 3.2. Fingerprint Collection

Due to the complexity of the indoor environment, fingerprint collection is affected by numerous factors, which cannot accurately describe the relationship between space with location fingerprinting. The positioning model is analyzed from the number of RPs and RSS samples in this section.

**Figure 4:**
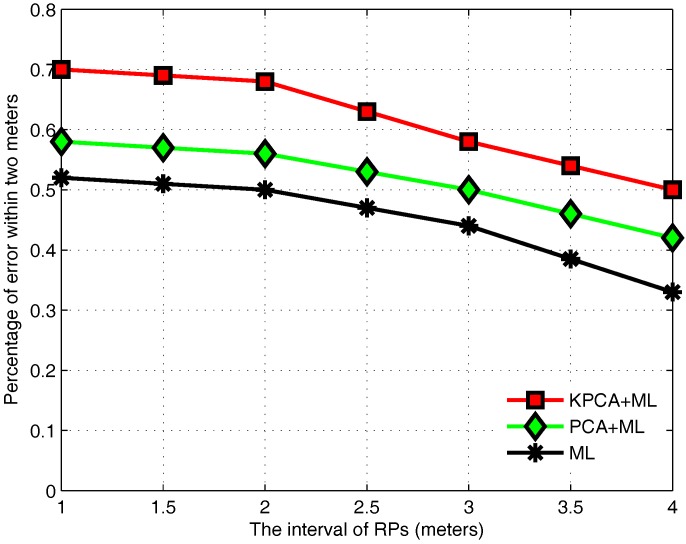
Effect of the interval of RPs.

Fingerprint collection usually consumes much power and time in the offline stage, so it is necessary to reduce the difficulty of the collection. Figure 4 reports the effect of the interval of RPs. It shows that the positioning accuracy is not significantly reduced when the interval of RPs is increased from 1 m to 2 m. When the interval continues increasing, the positioning error increased significantly. Therefore, it can be concluded that the number of RPs has a certain impact on the positioning accuracy. This stems from the fact that the larger the interval, the less the number of reference points. It cannot accurately describe the characteristics in an indoor environment. However, RPs without quantitative restriction will increase the calculation burden. To reduce the workload of fingerprint collection, the interval of RPs in this experiment is set to 2 m, since the interval between 1 m and 2 m does not have a significant effect on the positioning accuracy.

**Figure 5:**
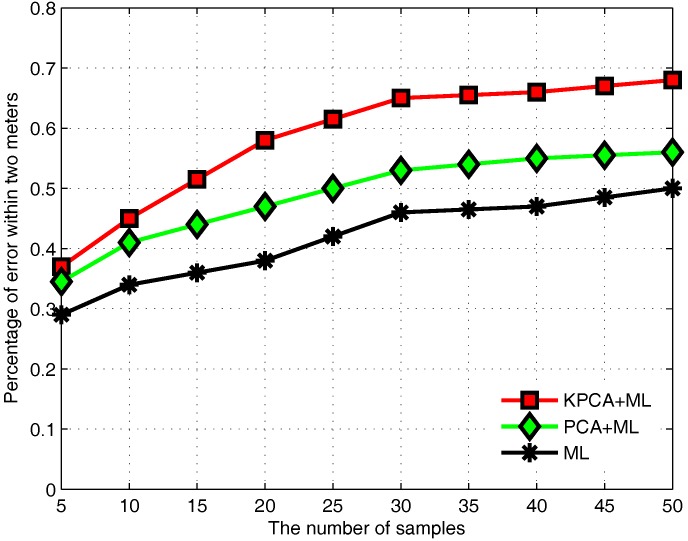
Effect of the number of samples.

In the offline phase, we need to collect a large number of RSS values to describe the relationship between location and fingerprint data. Selecting an appropriate number of samples not only can make the RSS signal more stable, but also directly affects the positioning accuracy of the positioning system. As shown in Figure 5, the trend of the percentage of positioning error within 2 m changing with the number of samples at each RP is reported. It can be found that when the number of samples collected at each RP is small, the accuracy of positioning increases as the number of samples increases. When the number of increasing samples is greater than 30 at each RP, the positioning accuracy of the positioning system tends to be stable.

### 3.3. Stable AP Selection

The AP selection algorithm proposed in this paper is based on the stability of the signal. APs that have higher stability can be effectively chosen to enhance the fingerprint’s stability and reliability. The stable AP selection is employed for fingerprint acquisition and online positioning. MaxMean [27] is the most popular AP selection algorithm, which selects APs whose average RSS values are large. To embody the advantages of the algorithm proposed, this section compares the stable AP selection with MaxMean.

**Figure 6:**
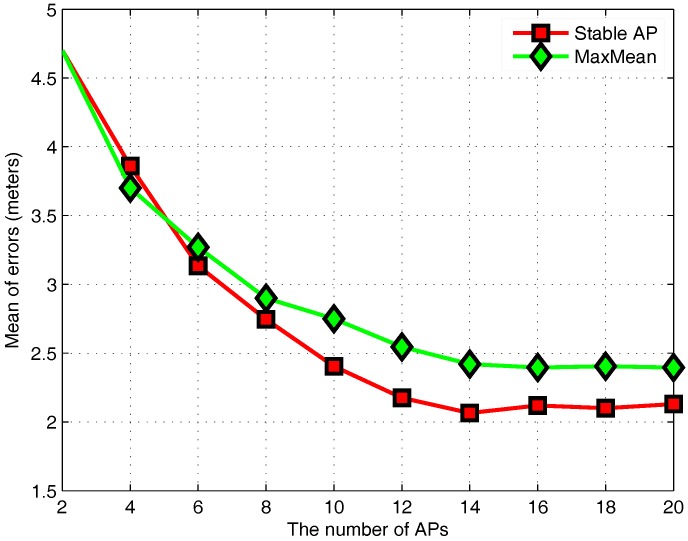
Effect of the number of APs.

To determine optimal AP numbers in different AP selection algorithms, Figure 6 compares the mean of positioning errors for MaxMean and stable AP selection in the case of different AP numbers. In theory, keeping the AP number as high as possible at each RP can accurately describe the characteristics in the current space, so the positioning accuracy should be higher. However, when the number of APs is small, the average positioning error decreases as the number of APs increases. When the number of APs reaches a certain value, the positioning accuracy tends to be stable. This is because some APs have no benefits for positioning. Therefore, it is necessary to remove redundant APs by the AP selection algorithm.

**Figure 7:**
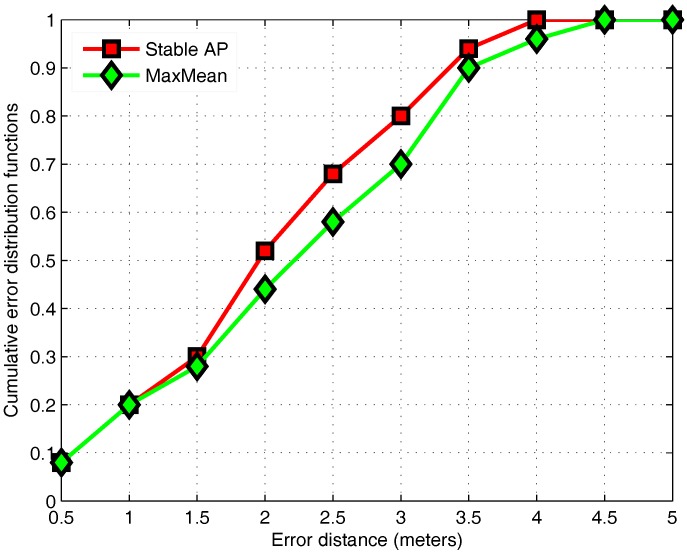
The CDF of the localization error of different AP selection algorithms.

MaxMean and stable AP selection can achieve the best positioning performance with 16 APs and 14 APs. Figure 7 shows the Cumulative Distribution Function (CDF) of the localization error with the optimal number of APs for each algorithm. The effect of MaxMean behaves worse than the stable AP algorithm. The error within 3 m of MaxMean is 70%, whereas our approach achieves 80 percent. MaxMean is used to select APs with larger average values, but it also means that the range of RSS values may become larger, so the variance of APs is large. Taking into account the stability of different APs, the proposed algorithm reduces the unreliability of fingerprint data and improves the positioning accuracy.

### 3.4. KPCA Algorithm

**Figure 8:**
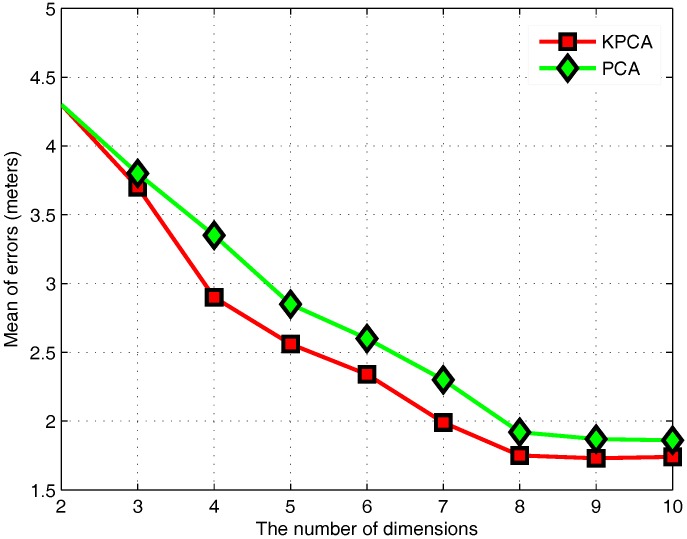
Effect of the number of dimensions.

**Figure 9:**
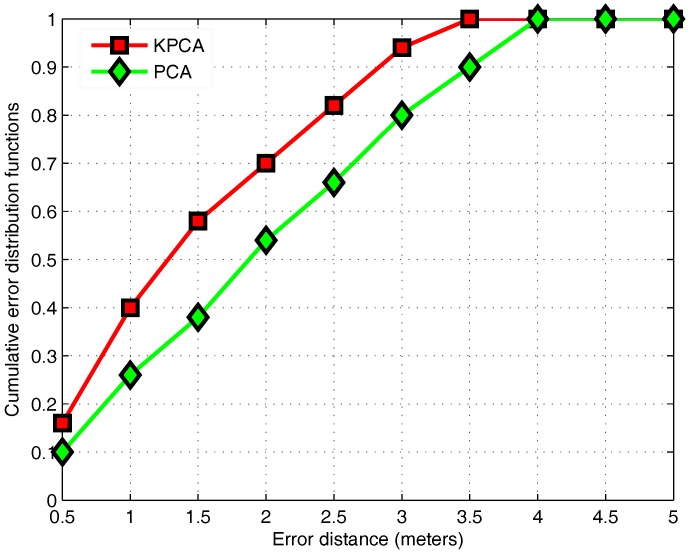
The CDF of the localization error of KPCA and PCA.

After the AP selection algorithm, the retained fingerprints are extracted to achieve a better positioning result. In this paper, the PCA algorithm based on the kernel function is used to analyze the nonlinear relationship of fingerprint information. The fingerprint in the low-dimensional space is mapped to the high-dimensional space by KPCA. Additionally, the feature set is selected to store in the fingerprint data. PCA only analyzes the linear relationship to remove the noise and redundancy. In this section, PCA is compared with the KPCA in Figure 8 and Figure 9.

To determine the optimal dimensions selected by two feature extraction algorithms, Figure 8 compares the mean of positioning errors for PCA and KPCA in the case of different dimensions. PCA is transformed into the low-dimensional space, so the highest dimension is the original data dimension. However, KPCA is implemented in the high-dimensional space, so the highest dimension after KPCA is the number of samples. However, it does not mean that the dimension after KPCA is higher than the original dimension, because the data for the lower dimension may already contain the vast majority of the information. From the perspective of the average positioning error, Figure 8 shows that PCA and KPCA both achieve the best positioning with eight dimensions.

Figure 9 shows the CDF of the localization error of the positioning model with the optimal dimensions for each algorithm. The KPCA algorithm has better performance than PCA. The probability of the PCA algorithm is 80 percent in error within 3 m. However, the KPCA algorithm has a 94 percent probability within 3 m. Moreover, it is observed that the mean of errors is improved by 14 percent while applying KPCA. Due to the complexity of the indoor environment, there is a large correlation between the RSS values. PCA can only analyze the linear relationship between the RSS. However, considering a nonlinear relationship, KPCA reduces the unreliability of the fingerprint data and improves the positioning accuracy.

According to Equation (17), the selection of the kernel parameter has an important influence on the positioning performance of this system. When σ is too small, the kernel function will decrease rapidly. It is difficult to identify the fingerprints that have high similarity. When σ is too large, fingerprints that have low similarity are hard to distinguish. There is no uniform method to determine the value σ, so the optimal value can be only obtained through the experiment. Table 1 shows the average error of the positioning system when the Gaussian kernel function selects different kernel parameters.

### 3.5. APC Algorithm

**Figure 10:**
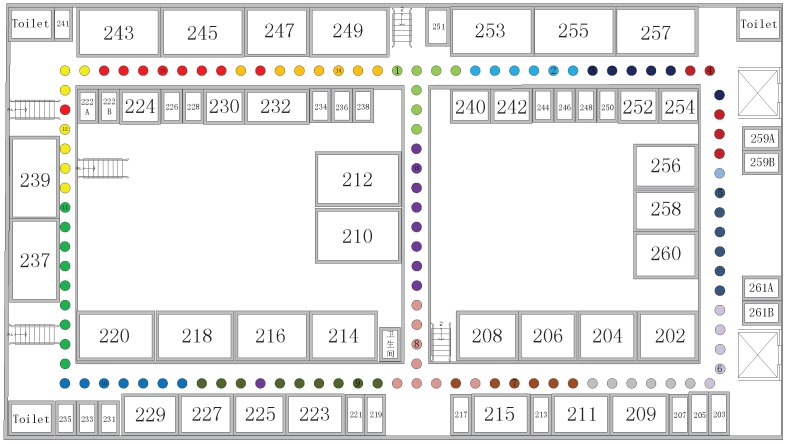
Clustering result.

According to Equation (20), different values of γ will yield different clustering results. When γ is small, the number of the clustering is large, and the change of γ will lead to a large change of the clusters’ number. The increase in the number of clusters helps to reduce the searching range and computational complexity. However, when the number of clusters is large, the similarity between adjacent classes will increase, which may lead to the failure of class matching. Therefore, it is indispensable to select an appropriate value of γ in the offline phase to form an appropriate number of clusters. Figure 10 shows the result of clustering when γ is equal to 0.3. The number of clusters is 15. Each point represents an RP. Different colors represent different clustering results, and there are some outliers in the corner. Nodes with numbers represent cluster centers. 

**Figure 11:**
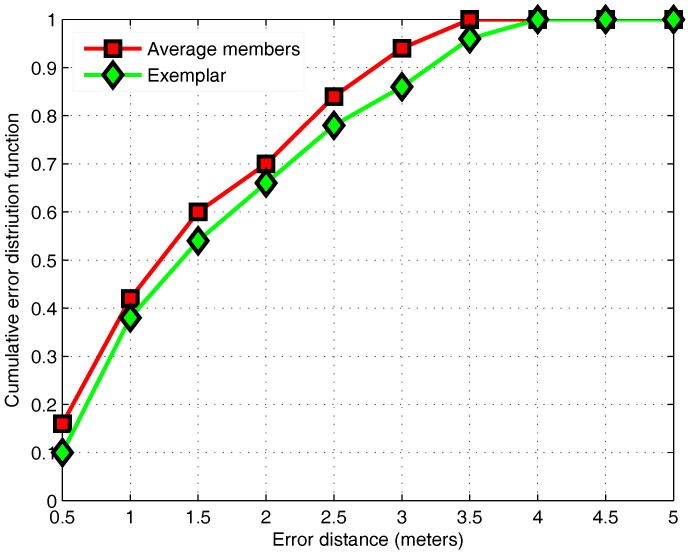
The CDF of the localization error of the different matching methods.

We provide two matching methods in the class-matching section. Figure 11 shows the positioning error of two matching methods. Because some clustering centers are at the edge of the class, it is best to calculate the mean of all class members as a matching sample.

### 3.6. Performance Evaluation

**Figure 12:**
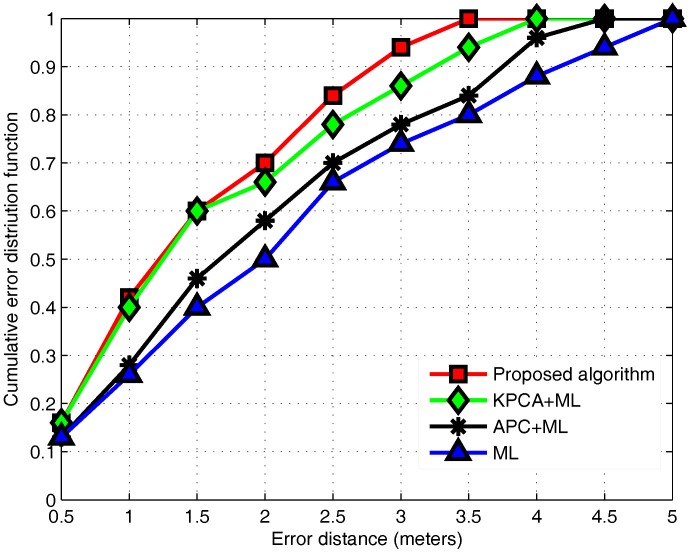
The CDF of the localization error of different algorithms.

We compare the methods used in this paper with fingerprinting approaches, known as the KPCA-ML, APC-ML and ML methods. We compare the performance of four methods from a CDF perspective. We find that the most primitive ML has the maximum error, and other methods have significant improvements compared with ML. The localization algorithm proposed in this paper uses KPCA to process original data, reducing the correlation among the data from different APs. Then, the APC algorithm is used to classify RPs to narrow the localization area. Finally, the ML estimation algorithm is used to achieve precise location. As shown in Figure 12, the proposed algorithm achieves a 94% probability of location accuracy within 3 m and a 38% improvement over the ML estimate alone.

## 4. Conclusions

In this paper, we propose a localization algorithm-based WLAN location fingerprinting for ensuring the high positioning accuracy and reducing energy consumption. Firstly, the improved AP selection algorithm is introduced based on the stability of signals to select the optimal subset of APs as the fingerprint data. It is verified by the tests that the accuracy of indoor positioning is improved because worthless APs are discarded.

Secondly, KPCA is proposed for nonlinear feature extraction. It can be used for eliminating redundancy and noise, reducing computational complexity and enhancing the positioning accuracy. KPCA is applied for mapping the low-dimensional data to high-dimensional, which can further increase the positioning performance compared to the traditional linear algorithm.

Finally, the indoor positioning model based on clustering and blocks is studied. The APC algorithm is employed to divide the positioning environment into various areas and to ensure the target area. The exact location of the target in each small area is determined. It also enhances the positioning accuracy with the computational complexity reduction.

Although this paper has enhanced the indoor location algorithm based on fingerprinting, there are some aspects deserving further discussion. Our algorithm is proposed only in a relatively simple flow of people under the test environment, which cannot cover particular indoor circumstances. In addition, the positioning system designed in this paper is only used to complete initial simple positioning. We will continue improving the positioning system to adapt to indoor environments and to make indoor positioning serve more people.

## Figures and Tables

**Table 1:** Effect of the kernel parameter.

kernel Parameter	σ = 0.2	σ = 0.4	σ = 0.6	σ = 0.8	σ = 1
the mean of errors	2.36	1.94	1.76	2.03	2.15

## Abbreviations

The following abbreviations are used in this manuscript:

RSS	Received Signal Strength
APs	Access Points
RPs	Reference Points
KPCA	Kernel Principal Component Analysis
APC	Affine Propagation Clustering
ML	Maximum Likelihood
LBSs	Location-Based Services
5G	the Fifth Generation Mobile Communication System
WLAN	Wireless Local Area Network
GPS	Global Position System
IR	Infrared
US	Ultrasound
RFID	Radio Frequency Identification
TOA	Time-Of-Arrival
AOA	Angle-Of-Arrival
KNN	K-Nearest-Neighbor
WKNN	Weighted K-Nearest Neighbor
PCA	Principal Component Analysis
CDF	Cumulative Distribution Function
